# Intestinal homeostasis: a communication between life and death

**DOI:** 10.1186/s13578-020-00429-9

**Published:** 2020-05-19

**Authors:** Lingyu Bao, Bingyin Shi, Yun-Bo Shi

**Affiliations:** 1 Department of Endocrinology, The First Affiliated Hospital of Xi’an Jiaotong University School of Medicine, No.277, Yanta West Road, Xi’an, Shaanxi, 710061 People’s Republic of China; 2 Section on Molecular Morphogenesis, Eunice Kennedy Shriver National Institute of Child Health and Human Development (NICHD), National Institutes of Health, Bethesda, MD 20892 USA

**Keywords:** Adult organ specific stem cell, Cell proliferation and differentiation, Apoptosis, Organ homeostasis, Cell-ECM interaction

## Abstract

Organ homeostasis is essential for organ physiology and disease prevention. In adult vertebrates, the intestinal epithelium is maintained through constant cell proliferation in the crypt and apoptosis of differentiated epithelial cells, mainly at the tip of the villus. Based on studies with altered cell proliferation and tissue damage in the adult mouse intestine, we hypothesize that there is a communication between cell proliferation in the crypt and cell death on the villus, likely via cell–cell and cell-ECM (extracellular matrix) interactions, to coordinate the rate of cell proliferation and death, thus ensuring epithelial homeostasis.

Adult organ homeostasis is essential for maintaining normal organ function throughout adult life in vertebrates. This is mainly accomplished through regulated proliferation of adult organ specific stem cells and their subsequent differentiation to replace lost or damaged cells. Proper coordination of cell loss/death and proliferation is essential to ensure organ maintenance and prevent organ overgrowth or tumor development.

One of the most-studied organs for tissue homeostasis is the adult vertebrate intestine. The adult intestinal epithelium performs the main functions of the intestine. Throughout adult life, the epithelium is constantly self-renewed. In mammals, the epithelium turnover time is about 1–6 days [1, 2], while in the metamorphosing anuran *Xenopus laevis*, the epithelium is replaced once every 2 weeks in the adult [3]. This epithelial self-renewal takes place through the proliferation of stem cells present near the bottom of the epithelial folds in anurans or in mammalian intestinal crypts (Fig. 1). As the daughter cells migrate along the epithelial fold or villus/crypt axis, they differentiate into different types of epithelial cells and eventually dies via apoptosis near the top of the fold or tip of the villus (Fig. 1), consequently completing the self-renewing cycle and maintaining tissue homeostasis.

**Fig. 1:**
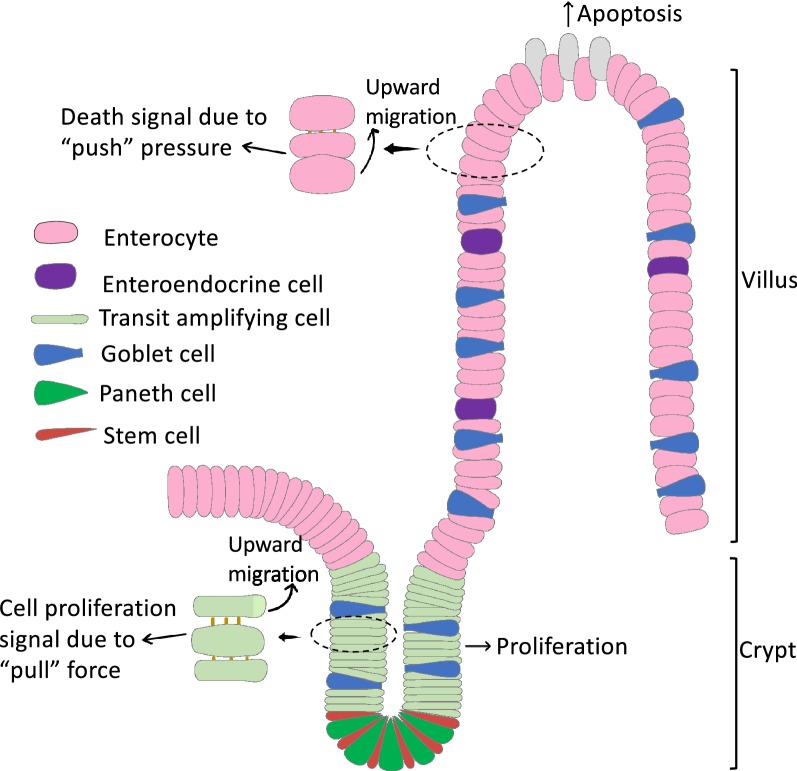
Schematic diagram of crypt-villus unit in the adult mammalian intestine and a model for the communications between cell death on the villus and cell proliferation in the crypt. Cell proliferation in the crypt leads to cell migrating toward the tip of the villus where most cell death occurs. The migrating cell will exert a”push” pressure on the adjacent cell above, e.g., the middle cell in the enlarged area near the tip of the villus). This may cause changes in cell–cell and/or cell-EMC interaction to facilitate cell death. Conversely, cell death on the villus will enhance upward migration of the cells in the crypt to replace the dying differentiated epithelial cells on the villus. This creates a “pull” tension/force to the adjacent cell below, e.g., the middle cell in the enlarged area near the crypt, thus leading to changes in cell–cell and/or cell-EMC interaction to facilitate cell proliferation

## Coordinated changes in cell proliferation and death to maintain homeostasis under pathophysiological conditions

For tissue homeostasis, cell proliferation and death need to be balanced. Thus, any alterations in one have to be countered by corresponding changes in the other. While this is generally assumed to be the case, few studies have analyzed both cell proliferation and death in the intestinal epithelium under altered pathophysiological conditions. In a recent study, we analyzed both cell proliferation and apoptosis in the adult intestinal epithelium by using a mutant mice model with a knockin mutation in the thyroid hormone receptor (TR) α gene locus that mimics human patients with TRα mutations [4]. Compared to wild type siblings, the heterozygous mutant mice had drastically reduced cell proliferation in the crypts, accompanied by a decrease in the apoptosis on the villi in the small intestine. On the other hand, the overall intestinal morphology was similar for the wild type and mutant mice, with only the intestinal villi slightly shorter, indicating that reduced cell proliferation was countered by the reduced cell death to maintain tissue homeostasis. Another example of reduced cell proliferation in the intestinal crypts is aged mice relative to young mice [5]. Although cell death was not analyzed in this study, the authors found that in the aged mice, the migration of the cells after proliferation was slower in the aged mice, reflecting reduced cell death at the tip of the villus, which therefore compensated for the reduced cell production in the crypts of aged mice compared to young mice.

Changes in the rate of apoptosis of differentiated epithelium can also lead to corresponding changes in cell proliferation in the crypt. The best examples here are intestinal regeneration for both small and large intestines after chemical or irradiation induced intestinal damage. Indeed, low dose radiation induces damages mainly on the villi of small intestine [5, 6]. To regenerate the epithelium, cell proliferation is drastically induced in the crypts [5, 6]. Once regeneration is complete, cell proliferation returns to normal rate.

## A simple model for cell–cell communication between cell death and proliferation

To maintain tissue homeostasis under altered pathophysiological conditions, it is critical to have prompt compensatory changes in cell proliferation and cell death. This requires a communication between dying and proliferating cells, often located far from each other, e.g., the dying cells mainly at the tip of the intestinal villus while the proliferating cells in the crypts. We propose a simple model based on the tight cell–cell contacts between adjacent epithelial cells and changes in cell-ECM (extracellular matrix) interactions as the cells migrate along the villus-crypt axis (Fig. 1). We hypothesize that cell migration up the villus will create a “pull” tension on the crypt cells due to the tight junctions between two adjacent epithelial cells, accompanied by altered interactions between the epithelial cell and basement membrane, the ECM underlying the epithelium. This “pull” tension and the altered cell-ECM interaction in turn signals the crypt cells, particular the transit amplifying cells, to proliferate. This would allow the crypt to quickly respond to epithelial damage since cells will migrate up the villus to cover the wounded epithelium.

Conversely, if cell proliferation increases due to genetic changes or other causes, there will be increased cell migration up the villus, thus exerting a “push” pressure on the differentiated cells on the villus, particularly at the tip, where cell death normally takes place (Fig. 1). This “push” pressure and accompanied changes in cell-ECM interaction may thus increase cell death. For example, disruption of epithelial cell attachment to the ECM if a cell is being forced out toward the lumen will likely lead to apoptosis as epithelial cell survival depends on cell-ECM interaction [7–10]. Such a model would also explain reduced cell death on the villus of the TRα knockin mutant intestine since cell proliferation decreases in the crypt [4]. The model further predicts that conditions that increase cell proliferation in the crypt will lead to more epithelial cell death on the villus.

## Conclusion

Tissue homeostasis is critical for organ function and disease prevention. A balance between cell removal and its replacement through cell proliferation is thus critical for adult organs. This requires a communication between cell death and cell proliferation. The propagation of physical tension via direct cell–cell interaction or ECM offers a fast and direct means of communication between cells separated by long distance as in the case of the tip of villus vs crypt in the adult intestine. Clearly, it would be interesting to test this experimentally.

## Data Availability

Not applicable.

## References

[1] MacDonald WC, Trier JS, Everett NB (1964). Cell proliferation and migration in the stomach, duodenum, and rectum of man: radioautographic studies. Gastroenterology.

[2] van der Flier LG, Clevers H (2009). Stem cells, self-renewal, and differentiation in the intestinal epithelium. Annu Rev Physiol.

[3] McAvoy JW, Dixon KE (1977). Cell proliferation and renewal in the small intestinal epithelium of metamorphosing and adult Xenopus laevis. J Exp Zool.

[4] Bao L, Roediger J, Park S, Fu L, Shi B, Cheng SY, Shi YB (2019). Thyroid hormone receptor alpha mutations lead to epithelial defects in the adult intestine in a mouse model of resistance to thyroid hormone. Thyroid.

[5] Nalapareddy K, Nattamai KJ, Kumar RS, Karns R, Wikenheiser-Brokamp KA, Sampson LL, Mahe MM, Sundaram N, Yacyshyn MB, Yacyshyn B, Helmrath MA, Zheng Y, Geiger H (2017). Canonical wnt signaling ameliorates aging of intestinal stem cells. Cell Rep.

[6] Metcalfe C, Kljavin NM, Ybarra R, de Sauvage FJ (2014). Lgr5 + stem cells are indispensable for radiation-induced intestinal regeneration. Cell Stem Cell.

[7] Ishizuya-Oka A, Hasebe T, Shi YB (2010). Apoptosis in amphibian organs during metamorphosis. Apoptosis.

[8] Fu L, Ishizuya-Oka A, Buchholz DR, Amano T, Matsuda H, Shi YB (2005). A causative role of stromelysin-3 in extracellular matrix remodeling and epithelial apoptosis during intestinal metamorphosis in Xenopus laevis. J Biol Chem.

[9] Su Y, Shi Y, Stolow M, Shi Y-B (1997). Thyroid hormone induces apoptosis in primary cell cultures of tadpole intestine: cell type specificity and effects of extracellular matrix. J Cell Biol.

[10] Werb Z, Sympson CJ, Alexander CM, Thomasset N, Lund LR, MacAuley A, Ashkenas J, Bissell MJ (1996). Extracellular matrix remodeling and the regulation of epithelial- stromal interactions during differentiation and involution. Kidney Int Suppl.

